# Assessing Motivational Factors in Young Serbian Athletes: A Validation Study of the Sport Motivation Scale-II

**DOI:** 10.11621/pir.2024.0301

**Published:** 2024-09-15

**Authors:** Damjan Jakšić, Jovana Trbojević Jocić, Marko Manojlović

**Affiliations:** a *University of Novi Sad, Serbia*; b *University of Kragujevac, Serbia*

**Keywords:** SMS-2, motivation, youth sports, adolescence, handball, volleyball, basketball

## Abstract

**Background.:**

Motivation is considered crucial in sports participation and performance, influencing athletes’ psychological well-being, investment in training, and interpersonal relationships. Self-determination theory (SDT) is a prominent framework used to understand motivation in sports, highlighting the importance of autonomous motivation for optimal performance and well-being. A large number of questionnaires for examining processes in sports were created by relying on the constructs of SDT.

**Objective.:**

This study explores the psychometric characteristics (construct validity) of the Sport Motivation Scale II (SMS-2), as well as gender and age differences in motivation among young Serbian athletes. This questionnaire has proven to be important for understanding the motivation of adult athletes, but so far, its psychometric characteristics have not been sufficiently examined on a sample of young athletes in Serbia. Given the high dropout rate from sports in adolescence, valid questionnaires to assess the motivation of young athletes can help to identify athletes who are at risk of leaving a sport.

**Design.:**

The sample consisted of 365 young athletes (51% girls, aged 12–16) from Serbia participating in team sports (at an organized level, not a recreational level), including volleyball, basketball, and handball. They completed the SMS-2 using paper and pen, in the presence of a psychologist and with parental consent obtained by the clubs. The questionnaire has been translated into Serbian. Young athletes from team sports were selected due to the large number of adolescents in Serbia who are engaged in organized team sports. There is a small number of adolescents who are involved in organized individual sports.

**Results.:**

Factor analysis of the SMS-2 revealed six factors, in line with the original structure of the Scale: identified motivation, intrinsic motivation, amotivation, external motivation, integrated motivation, and introjected motivation. The questionnaire demonstrates satisfactory psychometric properties, with Cronbach’s alpha coefficients indicating good internal consistency. Gender differences were obtained only in external motivation, where boys scored higher than girls. No significant differences emerge in motivation across age groups or among athletes participating in different sports. It is possible that differences were not found in relation to the type of sport because there are similarities in the process of working with young athletes in team sports.

**Conclusion.:**

The Sport Motivation Scale II (SMS-2) showed satisfactory psychometric characteristics in a Serbian sample of young athletes. The original structure was replicated, with six factors representing six types of motivation, in line with SDT. These findings suggest the SMS-2’s validity across gender, age, and sport types, offering a valuable tool for assessing motivation in young athletes engaged in organized team sports.

## Introduction

Motivation is one of the most frequently investigated factors in sports participation and performance; thus, it is not surprising that a large number of studies connect motivation with staying in sports (e.g., Trbojević & Petrović, 2021), the psychological well-being and mental health of athletes (Sheehan et al., 2018), the degree of investment in the training process (Pope & Wilson, 2012), sports results (Gillet et al., 2010), cognitive processes such as coping (Mouratidis & Michou, 2011), and the quality of interpersonal relationships (Chang et al., 2020). Research into motivation is still a current and important topic for a better understanding of both success in sports and the process within the athletes themselves.

### Motivation for Sports

The broadest understanding of motivation is that it is an activation process: initiating, directing, and regulating a person’s activities towards a certain goal (Vallerand & Thill, 1993). Considering that motivation is a latent variable, its measurement is, therefore, a complex task.

When creating a questionnaire for assessing sports motivation, the authors started from different theoretical positions, such as Self-Efficacy Theory (Bandura, 1997) and Achievement Goal Theory (Ames, 1992; Nicholls, 1989). However, in recent years, one of the most frequently applied and examined theories is Self-Determination Theory (SDT, Deci & Ryan, 1985; Ryan & Deci, 2017), which has also found application in the sports context.

SDT is a macro theory that observes human processes from the viewpoint of volition and autonomy, and asserts that if we approach an activity with the experience of choice and autonomy, we will be more successful in its performance — i.e., the more autonomous, more willing our motivation is, the more successful we will be and the better our psychological well-being. SDT deals explicitly with the issue of motivation as a multidimensional construct that extends on a continuum from amotivation (when an individual has no desire and intention to participate in an activity) through external motivation (when an individual is active only because of the external value that the activity brings) to intrinsic motivation (participation for satisfaction) (Trbojević & Petrović, 2021). According to SDT, the individual tries to integrate and organize values, regulatory processes, and experiences from the environment into the self (Weinstein & DeHaan, 2014). The tendency towards integration takes place within the individual over time, but also between the individual and others. The goal of integration is the formation of autonomous self-regulation that entails internalized personal values, beliefs, and interests that encourage voluntary actions (Weinstein & DeHaan, 2014).

SDT distinguishes six types of motivation that differ in relation to the level of autonomy and control, such as amotivation, external motivation, introjected motivation, identified motivation, integrated motivation, and internal motivation.

*Amotivation* is the absence of motivation due to low self-regulation or autonomy; the experience of incompetence mainly accompanies it. In the context of sports, athletes with amotivation feel incompetent and do not know why they play sports.

Concerning *external motivation*, behaviour is regulated by external factors like reward and punishment, and the athletes, for example, play sports to win a medal or to avoid criticism.

*Introjected motivation* represents behaviour regulated by internal contingencies of self-esteem and self-regard, i.e., intrapersonal rewards and punishments that motivate action and performance efforts (Ryan et al., 2023). Thus, athletes with introjected motivation participate in sports activities because they would feel bad if they did not set aside time for them. In addition, athletes feel they must compete in sports, and not because they want to. Athletes also engage in activities to increase their self-confidence and improve their self-image based on external factors, because the values are not internalized.

*Identified motivation* implies that an individual behaves in a way that will enable the achievement of a relevant goal (Lonsdale et al., 2009). This type of regulation is more autonomous than external and introjected motivation. It implies, before internalization, an evaluation of a goal or value. Internalization will occur if the goal or value is assessed as relevant to the individual. Athletes with identified motivation engage in sports to develop certain parts of their personality that they consider important.

*Integrated motivation* is to the greatest extent autonomous and self-regulated compared to other forms of motivation. Integrated motivation implies that regulations are assimilated with the self and are integral to beliefs based on personal needs (Ryan & Deci, 2000).

*Internal motivation* refers to participation in activities that spontaneously lead to a reward and new knowledge, causing enjoyment and satisfaction of basic needs. This type of motivation is autonomous, because a person’s behaviour is based on initiative with a high degree of satisfaction, enjoyment, and conscious selection of activities.

The specificity of sports is reflected in the fact that a person cannot be successful without training, and according to the theory of deliberate practice (Ericsson et al., 1993), a larger number of deliberate practice hours is necessary to be successful, rather than innate talent. Deliberate practice involves effortful activity that is closely connected with motivation — person needs to be motivated to practice for hours and hours, which are aimed at improving one’s performance, but are sometimes not so enjoyable, or do not lead directly to rewards (Rottensteiner et al., 2013).

Research has recognised different types of motivations for playing sports in relation to the training process, competition, and type of sport. Thus, motivation for achievement is more pronounced during competitions, and internal forms of motivation that are focused on investing effort and learning are more prevalent during the training process, in athletes in both individual and team sports (van de Pol & Kavussanu, 2012). Young athletes, who are still at the stage of sports specialization and have not progressed to the stage of professional sports, achieve slightly higher scores on intrinsic motivation (Rottensteiner et al., 2015) than athletes who play sports professionally (Stewart & Meyers, 2004).

Athletes’ motivation during adolescence comes through a series of changes — the training system itself changes, there is more training and competition, with greater scope — all of which can affect the process and stability of motivation. Bearing in mind that motivation is one of the factors in further sports participation and achievement in sports, testing the motivation of young athletes is especially important with the aim of overcoming the negative consequences of amotivation both for performance and retention, as well as for the mental health and well-being of young athletes.

### Testing Motivation for Sports

The instruments created so far are mostly self-report questionnaires; six questionnaires are most often used in sports psychology, which aim to assess the motivation of athletes (Clancy et al., 2017): the Sport Motivation Scale (Pelletier et al., 1995), the Intrinsic Motivation Inventory (McAuley et al., 1989), the Situational Motivational Scale (Guay et al., 2000), the Perceptions of Success Questionnaire (Roberts et al., 1998), the Behavioral Regulation in Sport Questionnaire (Lonsdale et al., 2008), and the Task and Ego Orientation in Sport Questionnaire (Duda, 1989).

Guided by the principles of SDT, in 1995, French researchers created the Sport Motivation Scale (Pelletier et al., 1995), one of the most frequently used questionnaires, which was translated and implemented among athletes from various countries. The scale aims to measure different types of motivation for sports. The scale consists of 28 items that form intrinsic motivation subscales (IM-to know, IM-to accomplish, IM-to experience), extrinsic motivation (identified regulation, introjected regulation, external regulation), and amotivation.

The authors of the scale decided to revise it, and in 2013, they created the Sport Motivation Scale II (SMS-2, Pelletier et al., 2013). The need for a revised version of the questionnaire arose because the original scale was not fully in line with the SDT, so that both the metric characteristics and the factor structure of the questionnaire oscillated when applied in different countries and at various ages (Pelletier et al., 2013). To improve certain items, the authors added a subscale of integrated motivation and shortened the scale to make its use easier and faster (Pelletier et al., 2013).

The revised scale consists of 18 items and measures six types of motivation defined according to SDT. The fit indices ranged from satisfactory to very good (RMSEA = .07; RMSEA 90% CI = .05-.08; CFI = .94; NFI = .90; TLI = .92), while the item factor loadings ranged between .47 and .95 (Pelletier et al., 2013).

Available studies have largely examined the factor structure and psychometric characteristics of the original scale for motivation for sports (e.g., Bayyatet al., 2016; Komarc et al., 2020; Mladenović & Stojanović, 2022), and research on the revised scale is somewhat scarce, but current. The revised SMS-2 scale was translated into French (Pelletier et al., 2019), Chinese (Li et al., 2018), Turkish (Ocal & Sakalli, 2018), Spanish (Granero-Gallegos et al., 2018), and Portuguese (Junior et al., 2014; Rodrigues et al., 2021). On a sample of Turkish athletes in various sports (individual and collective), SMS-2 proved to be a good six-factor solution, where Cronbach’s alpha for the total scale was .76 and .72 for intrinsic, .61 for integrated, .81 for identified, .55 for introjected, .73 for external, and .72 for amotivation (Ocal & Sakalli, 2018). The Persian adaptation of the SMS-2 also proved to be valid, with the original six-factor solution and good internal consistency (intrinsic = .80, integrated = .78, identified = .77, introjected = .75, external = .77, amotivated = .80, and the total = .79) (Kashani, 2016).

The validity of the scale was examined primarily in a population of athletes, who are often students who attend faculties for sports science. One of the few available studies that examined a sample of younger athletes (from 16 to 21 years of age) engaged in various individual and team sports was conducted in Malaysia (Chin et al., 2021), where the proposed six-factor solution of the SMS-2 was obtained, but with somewhat weaker internal validity: .71 intrinsic, .73 integrated, .75 identified, .46 introjected, .61 external, and .52 amotivation.

When examining the motivational profile of young athletes of both sexes in team sports, based on questionnaires based on Self-Determination Theory and Achievement Goal Theory, researchers found that the largest number of young athletes, about 36%, belong to the category of athletes in whom the autonomous form of motivation and the controlling form of motivation are equally expressed, and 28% belong to the category of highly expressed and autonomous and controlling motivation (Rottensteiner et al., 2015).

Most of the studies that explored gender differences in the motivation for sports either included recreational sports, were conducted on a student population, or applied questionnaires that did not refer so much to internal motivation processes, but were more focused on defining different motives such as social motives, competition motives (*e.g*., Malčić, 2012), or goal orientation. Such studies have generally found that women gravitate toward social and affiliative motives for playing sports and men more toward competitive motives and ego orientation (*e.g.*, Flood & Hellstedt, 1991; Murcia et al., 2007). In addition, it should also be noted that men score higher on intrinsic and extrinsic motivation to engage in physical activity than women (*e.g.*, Sáez et al., 2021). In the adolescent population, some research found that boys and girls differ in motives for physical activity, where boys achieve higher scores on motives such as socializing, competition, enjoyment, social recognition, and strength and persistence, and girls on motives like appearance, agility, maintaining and improving health, and body mass control (Ivanović & Ivanović, 2018). Regarding investigations that applied the SMS-2, gender differences were not recorded in most types of motivation for playing sports, but it was found that males achieved higher scores on extrinsic motivation than females — i.e., that girls achieved the highest scores in intrinsic motivation and lower in extrinsic motivation than boys (Miller, 2000; Recours et al., 2004). Similar results were obtained in a study performed on a sample of adolescents from Norway. Girls achieved higher scores on intrinsic motivation for playing sports and boys on extrinsic motivation (Jakobsen & Evjen, 2018).

Scientific evidence with respect to the age differences in motivation is quite scarce. Studies pertaining to young athletes and adolescents were mostly conducted outside the framework of organized sports and more in the context of physical activity in general. Thus, research conducted on Greek adolescents examined age differences in the motivation for attending physical education classes, where older adolescents achieve lower scores on internal motivation (Digelidis & Papaioannou, 1999). A study conducted on athletes aged 11 to 19 revealed that self-determining motivation decreases with age — i.e., that the degree of autonomous motivation for playing sports decreases with age (Guzman and Kingston, 2012). The studies support the hypothesis (and experience of sports organizations) that adolescence is a risky age period for dropping out of sports due to a decrease in motivation for playing sports.

Just as research aimed at determining gender and age differences in motivation for playing sports among young athletes is rare, so is research that has dealt with the question of whether there are differences in motivation for playing sports in relation to the type of sport played at this age. Some studies have shown that athletes who play individual sports achieve lower scores on enjoyment as an internal motive for playing sports, compared to athletes who play team sports (Jakobsen, 2014), while some studies have obtained the opposite findings (Howard et al., 2018).

In terms of athletes in Serbia, studies were directed toward the examination of the original scale (e.g., Mladenović & Stojanović, 2022; Vesković, 2012), whereas until now, no research on the revised scale had been conducted on young Serbian team sports athletes. The topic of motivation among younger athletes is of particular importance, since adolescence is the period when the largest number of children drop out not only from physical activity, but also from organized sports (Trbojević & Petrović, 2021).

The aim of this research is to apply and validate the Sport Motivation Scale-II among young Serbian athletes, contributing to the comprehensive understanding of motivational factors in the context of sports engagement. Also, our aim is to examine gender and age differences in motivation, as well as differences in relation to the type of sport.

## Methods

### Participants

The sample consisted of 365 young athletes from Serbia, province of Vojvodina (51% girls) aged 12 to 16 years (mean = 13.79, SD = 1.25), who train in basketball (N = 131), volleyball (N = 125), and handball (N = 109). They trained three to five times a week (average of eight hours of training per week), 80% of them trained in only one sport at the time of the research. They all trained and competed at the club level in an organized manner. More details relating to the participants’ characteristics are provided in *Table 1*.

**Table 1:** Characteristics of the Sample

Variables	Girls	Boys	Total
Age category			
12 years	36	21	57
13 years	57	63	120
14 years	34	34	68
15 years	42	40	82
16 years	19	19	38
Type of sport			
Volleyball	65	60	125
Basketball	65	66	131
Handball	56	53	109

### Procedure

The first phase of the research included the preparation and translation of the questionnaire from English to Serbian by a sports psychologist. The author’s consent was previously obtained for the use of the questionnaire. During the translation, linguistic constructions were considered to make them understandable to adolescents.

The second phase of the research entailed establishing contacts with sports clubs of the Autonomous Province of Vojvodina in the domain of collective, indoor sports, including volleyball, basketball, and handball. A public invitation to participate in the research was sent to clubs from the territory of Vojvodina.

The third phase of the research involved data collection. The inclusion criteria to participate in this study were: (a) to be actively training in a sports club during the time of data collection; (b) to be actively training in the same sport for at least 1 year and at least 4 months in their current club; (c) to be actively training in sports such as volleyball, basketball, or handball; (d) to consent to participate in the study. Young athletes from team sports were selected due to the large number of adolescents in Serbia who are engaged in organized team sports, but also because of the high rates of adolescents who drop out of these three sports in Serbia. There is a small number of adolescents who are involved in organized individual sports, so the sample was based on the accessibility criterion.

Data was collected during 2017 in Vojvodina. Data collection was carried out on the premises of the clubs, on the field itself, or in the dressing rooms. Of note, the athletes were alone with the psychologist while filling out the questionnaire. The procedure had been previously explained to them, as well as that the data would not be publicly available but would be used strictly for scientific purposes, and that only the psychologist conducting the research would have access to their answers. The coaches were also informed that they would not have access to their athletes’ answers and were asked to leave the room while the athletes completed the questionnaire. Filling out the questionnaire took an average of 15 minutes. The working conditions were not at a high level due to the lack of adequate space when filling out the questionnaires, as well as the fact that some respondents filled out the questionnaires immediately after or immediately before training, which led to reduced motivation to work.

### Instruments

The Sport Motivation Scale-II (SMS-2, Pelletier et al., 2013) consists of 18 items that measure intrinsic motivation (*e.g*., “Because it is very interesting to learn how I can improve”), identified motivation (*e.g*., “Because I found it is a good way to develop aspects of myself that I value”), introjected motivation (*e.g*., “Because I would feel bad about myself if I did not take the time to do it”), integrated motivation (*e.g*., “Because practicing sports reflects the essence of whom I am”), extrinsic motivation (*e.g*., “Because people I care about would be upset with me if I didn’t”), and amotivation (*e.g*., “I used to have good reasons for doing sports, but now I am asking myself if I should continue”). In the original questionnaire, the athlete answered on a seven-point Likert scale to what extent a certain reason for playing sports applies to him or her. A five-point Likert scale was employed on the sample of the current study, since the questionnaire was filled out by young adolescents, for whom it turned out that the five-point scale was more comprehensible.

### Data Analysis and Research Design

The study was an instrumental study within empirical studies based on a quantitative methodology (Montero & León, 2007).

Data analysis was conducted on ordinal data, necessitating specialized statistical procedures. Spearman rank correlation was employed to assess the relationships between variables, with the resulting Spearman’s matrix serving as the foundation for subsequent factor analysis. It is noteworthy that the factor analysis was carried out using Promax rotation. Four distinct criteria, involving Kaiser-Guttman’s, Parallel, Optimal Coordinates, and Acceleration Factor Criteria, were employed to determine the significance of the identified factors. Importantly, the factor analysis was performed not on the raw matrix but on Spearman’s matrix, emphasizing the robustness of the analytical approach. The analysis was conducted using R 4.3.2, a language and environment for statistical computing (R Core Team, 2023). R is available from the R Foundation for Statistical Computing, Vienna, Austria (https://www.R-project.org/), leveraging libraries such as ggstatsplot, metan, ggcorrmat, corrgram, nFactors, and psych to ensure comprehensive and rigorous data exploration. Cronbach α coefficients were also calculated for inter-item reliability. For differences between gender, age, and sports, Mann-Whitney and Kruskall-Wallis tests were applied.

## Results

We present the results in the following order: First, we show the Spearman correlation matrix to illustrate the relationships between the ordinal variables. Next, we detail the results of the factor analysis, including the extraction of significant factors and their loadings. Following this, we report the outcomes of the Mann-Whitney U test to examine differences between genders. Subsequently, we present the Kruskal- Wallis H-test results to explore variations across different age groups and sports. This structured approach provides a comprehensive overview of the data and facilitates a clear understanding of the findings.

The Spearman rank correlation revealed a range from low to high associations among variables (*Table 2*). Principal components were identified, and the selection of significant components was based on consultation of four criteria.

**Table 2:** Spearman’s Intercorrelation Matrix

	1.	2.	3.	4.	5.	6.	7.	8.	9.	1.	11.	12.	13.	14.	15.	16.	17.	18.
1. SMS1		.038	.045	.002	.000	.001	.000	.000	.200	.718	.514	.000	.194	.010	.019	.000	.049	.033
2. SMS2	.106		.100	.000	.002	.602	.287	.000	.092	.000	.000	.505	.000	.004	.019	.388	.000	.701
3. SMS3	.102	-.084		.000	.630	.000	.031	.277	.000	.017	.001	.000	.029	.000	.383	.003	.000	.000
4. SMS4	.156	-.198	.202		.624	.004	.045	.754	.000	.000	.000	.084	.000	.000	.919	.000	.000	.035
5. SMS5	.288	.162	.025	.025		.008	.000	.000	.238	.003	.870	.044	.002	.907	.000	.000	.178	.292
6. SMS6	.167	-.027	.209	.147	.136		.000	.064	.000	.910	.002	.000	.693	.000	.002	.000	.001	.000
7. SMS7	.418	.055	.110	.103	.253	.202		.000	.021	.804	.149	.001	.113	.000	.000	.000	.145	.002
8. SMS8	.278	.204	.056	.016	.524	.095	.331		.559	.013	.019	.014	.006	.304	.000	.000	.008	.050
9. SMS9	.066	-.086	.385	.253	-.060	.197	.118	-.030		.015	.000	.000	.001	.000	.822	.000	.000	.000
1. SMS 10	.019	.449	-.122	-.225	.149	-.006	.013	.126	-.125		.000	.533	.000	.001	.148	.170	.000	.139
11. SMS11	.033	-.227	.169	.446	.008	.156	.074	-.120	.219	-.270		.004	.000	.000	.507	.000	.000	.002
12. SMS 12	.202	.034	.242	.088	.103	.464	.166	.126	.321	-.032	.147		.146	.000	.084	.000	.000	.000
13. SMS 13	.066	.361	-.111	-.233	.155	-.020	.081	.141	-.166	.535	-.317	-.075		.019	.064	.758	.000	.619
14. SMS 14	.131	-.148	.269	.285	-.006	.269	.192	.053	.335	-.168	.295	.278	-.120		.008	.000	.000	.000
15. SMS 15	.120	.120	-.045	.005	.310	.154	.178	.363	-.012	.074	.034	.088	.095	.135		.000	.491	.006
16. SMS 16	.312	.044	.151	.177	.239	.292	.376	.213	.278	-.070	.182	.293	-.016	.331	.233		.000	.000
17. SMS 17	.101	-.187	.308	.272	-.069	.177	.075	-.135	.530	-.234	.275	.210	-.263	.311	-.035	.290		.000
18. SMS 18	.109	-.020	.269	.108	.054	.577	.158	.100	.317	-.076	.161	.561	-.026	.338	.139	.390	.261	

Results from *Table 3* mostly indicate a six-factor solution, each factor formed by three items. Principal components are rotated in a better Promax solution, and factors saturated with more than .300 were taken into account in the interpretation. All results are in line with the proposed factors defined by the authors of Sport Motivation Scale-2: identified motivation (first factor), intrinsic motivation (second factor), amotivation (third factor), external motivation (fourth factor), integrated motivation (fifth factor), and introjected motivation (sixth factor).

**Table 3:** Pattern Matrix Based upon Correlation Matrix

Variable	RC1	RC4	RC3	RC2	RC6	RC5	h^2^	u^2^	com
SMS–18	**.883**	.064	–.008	–.003	–.077	–.074	.756	.244	1.04
SMS–6	**.869**	–.178	.011	–.032	.043	.038	.675	.325	1.10
SMS–12	**.780**	.094	–.034	–.034	–.170	.057	.627	.373	1.14
SMS–9	.008	**.857**	.081	–.029	.027	–.086	.701	.299	1.04
SMS–3	–.016	**.785**	–.050	.076	–.169	–.032	.535	.465	1.13
SMS–17	–.051	**.703**	–.071	–.107	.129	.012	.601	.399	1.15
SMS–10	–.012	.011	**.873**	–.035	.019	–.062	.730	.270	1.02
SMS–13	.018	–.068	**.776**	–.054	–.035	.073	.643	.357	1.05
SMS–2	–.037	.097	**.704**	.081	–.073	.014	.546	.454	1.09
SMS–15	.103	–.102	.034	**.829**	.190	–.311	.653	.347	1.47
SMS–8	–.081	.066	–.071	**.787**	–.206	.155	.708	.292	1.28
SMS–5	–.098	–.025	–.001	**.718**	–.023	.155	.600	.400	1.14
SMS–11	.008	–.084	–.065	–.019	**.835**	–.056	.680	.320	1.04
SMS–4	–.197	.059	.018	–.026	**.805**	.130	.641	.359	1.19
SMS–14	.224	.228	.035	.055	**.409**	–.013	.447	.553	2.26
SMS–1	–.028	–.066	.008	–.076	.022	**.882**	.698	.302	1.03
SMS–7	.026	–.057	–.019	.039	.031	**.773**	.622	.378	1.02
SMS–16	.245	.126	.041	.150	.180	**.348**	.483	.517	3.20
Cronbach’s α	.77	.70	.66	.68	.59	.64			

*Notes. RC = rotated component. h^2^ = squared multiple correlation. u^2^ = unique variance. com = communalities.*

The results indicate a strong relationship between the variables and the underlying factors, as evidenced by the h^2^ values (and their corresponding uniqueness values). These relationships range from 75.6% for SMS-18, which loads strongly on the first rotated component, to 44.7% for SMS-14, which shows a substantial association with the sixth factor.

Young Serbian athletes achieve above theoretical average score on more autonomous types of motivation — intrinsic motivation, integrated motivation (Table 4), but also on identified motivation.

**Table 4:** Motivation for Sports in Young Athletes

	N	Minimum	Maximum	Mean	Std. Deviation
Intrinsic Motivation	355	3.00	15.00	13.4535	1.90745
Integrated Motivation	350	5.00	15.00	12.8371	2.05910
Identified Motivation	349	3.00	15.00	12.4871	2.52527
Introjected Motivation	346	3.00	15.00	8.0867	3.26749
External Motivation	354	3.00	15.00	5.1582	2.59611
Amotivation	355	3.00	14.00	3.8085	1.73121

The results of the Mann-Whitney test, as shown in *Table 5*, indicate statistically significant differences between boys and girls regarding external motivation. In this aspect, boys had a median score of 5 and an interquartile range (IQR) of 3 to 7, compared to girls, who had a median score of 4 and an IQR of 3 to 6. This suggests that, on average, boys reported higher levels of external motivation than girls.

**Table 5:** Gender and Motivation for Sports

Factor	Boys (N = 179)	Girls (N = 186)			
	Median [IQR]	Median [IQR]	U	Z	p–value
Identified Motivation	13 [11–15]	13 [11–14]	15215.50	–1.44	.149
Intrinsic Motivation	14 [12–15]	14 [13–15]	15456.50	–1.23	.218
Amotivation	3 [3–4]	3 [3–3.25]	16148.00	–.64	.521
External Motivation	5 [3–7]	4 [3–6]	14236.00	–2.47	**.014**
Integrated Motivation	13 [11–15]	13 [12–14]	15821.50	–.83	.404
Introjected Motivation	8 [5–11]	8 [6–11]	16309.00	–.34	.736

*Notes. IQR = interquartile range. U = Mann-Whitney U-test. Z = z-value. p = significance.*

Although not statistically significant, there are variations in motivational aspects between boys and girls. For identified motivation, intrinsic motivation, and integrated motivation, the median scores were similar for both genders, with overlapping IQRs indicating no disparities. However, for introjected motivation, despite both boys and girls having a median score of 8, the IQR for girls (6 to 11) was slightly narrower than that for boys (5 to 11), suggesting a more consistent response trend among girls in this domain.

*Table 6* displays an analysis of what drives individuals to participate in sports across age groups. No statistically significant differences were revealed in motivation levels for sports among athletes of all the age brackets. The median scores for types of motivation, such as identified, intrinsic, amotivation, external, integrated, and introjected, show trends across the range of 12 to 16.

**Table 6:** Age and Motivation for Sports

Factor	12 years (N=57)	13 years (N=120)	14 years (N=68)	15 years (N=82)	16 years (N=38)	H	p-value
	Median [IQR]	Median [IQR]	Median [IQR]	Median [IQR]	Median [IQR]		
Identified Motivation	12 [11–14]	13 [11–15]	13 [11–14.75]	13 [11.75–15]	13 [11–15]	4.057	.255
Intrinsic Motivation	14 [12–15]	14 [13–15]	14 [12–15]	14 [11.75–15]	14 [13–15]	3.361	.339
Amotivation	3 [3–4]	3 [3–4]	3 [3–3]^D^	3 [3–4.25]^C^	3 [3–4.25]	4.331	.228
External Motivation	4 [3–7]	5 [3–6]	4 [4–5]	4 [3–6.25]	4.5 [3–7]	1.876	.599
Integrated Motivation	13 [11–14]	13 [12–14]	13 [12–15]	13 [11–15]	13.5 [12–15]	4.135	.247
Introjected Motivation	7 [5–9]	8 [6–10]	7 [5–11]	8 [6–11]	8.5 [5–11]	7.617	.055

*Note. H = Kruskal-Wallis H-test.*

Differences in motivation levels of athletes according to the type of sports are depicted in *Table 7.* The findings suggest that there are no differences in motivation for sports among athletes participating in team sports, like volleyball, basketball, and handball. The median scores for types of motivation exhibit similar trends across all three sports.

**Table 7:** Type of Sports and Motivation for Sports

Factor	Volleyball (N=125)	Basketball (N=131)	Handball (N=109)	H	p-value
	Median [IQR]	Median [IQR]	Median [IQR]		
Identified Motivation	13 [11–15]	13 [11–15]	13 [12–14]	.537	.765
Intrinsic Motivation	14 [13–15]	14 [12–15]	14 [13–15]	.713	.700
Amotivation	3 [3–4]	3 [3–4]	3 [3–3]	.475	.789
External Motivation	4 [3–7]	4 [3–6]	4 [3–6]	.620	.733
Integrated Motivation	13 [12–14.50]	13 [12–15]	13 [12–14]	.075	.963
Introjected Motivation	7 [5–10]	8 [6–11]	8 [6–11]	2.903	.234

*Notes. IQR = interquartile range. H = Kruskal-Wallis H-test.*

## Discussion

The phenomenon of motivation for sports has been a continuous research question through different generations of athletes, with a special focus on young athletes. Bearing in mind that there is a trend toward dropping out of sports in adolescence, when young athletes in collective sports move to the stage of sports specialization (Trbojević & Petrović, 2020), and soon after to the investment stage, which leads to the path of professional sports, it is necessary to study the factors that contribute to that trend. Most importantly, a robust body of evidence indicates that motivation for sports represents one of those factors. Therefore, this research aimed to determine the psychometric characteristics of one of the most frequently used questionnaires of motivation for sports in the world, the Sport Motivation Scale II – SMS-2 (Pelletier et al., 2013), on a sample of young Serbian athletes who play in team sports, such as volleyball, basketball, and handball.

The results of the factor analysis show that this questionnaire has satisfactory metric characteristics in the Serbian sample and that six factors of the questionnaire can be distinguished, which the authors themselves proposed, and many studies on other populations of athletes also obtained (Li et al., 2018; Ocal & Sakalli, 2018; Pelletier et al., 2019). Thus, on the Serbian sample of young athletes aged 12 to 16, we obtained a six-factor questionnaire solution with identical loading of items as in the original Scale: identified motivation (6, 12, 18); intrinsic motivation (3, 9, 17), amotivation (2, 10, 13), external motivation (5, 8, 15), integrated motivation (4, 11, 14), and introjected motivation (1, 7, 16).

Cronbach’s alpha for obtained factors was somewhere in line with previous research (*e.g*., Ocal & Sakalli, 2018): identified motivation (.77); intrinsic motivation (.70), amotivation (.66), external motivation (.68), integrated motivation (.59), and introjected motivation (.64). Factor integrated motivation had the lowest Cronbach’s alpha score in the sample of young athletes, which may be the result of the age of the athlete. Young athletes have a developmental task in adolescence to question themselves, “Who am I?”, to form an identity at the end of this developmental period. Having in mind that integrated motivation implies that regulations are assimilated with the Self, that they are an integral part of beliefs and based on personal needs (Ryan & Deci, 2000), it could be that young athletes have some difficulties understanding items that form this factor because they are in the process of questioning their personal Self and forming their identity. During data collection, some athletes asked for help clarifying these items.

The second goal of our research was to further investigate SMS-2 in line with gender, age, and sport type. The results show that there were no age differences in motivation for sports, and no differences in motivation in athletes who play different types of sports (volleyball, basketball, and handball). It is possible that there is a similar motivation for participation in team sports, and that differences were not obtained in relation to the type of sport because there are similarities in the process of training young athletes in these three sports. As to difference in motivation in relation to age, a small number of studies that focused on age differences in motivation in young athletes found that autonomous motivation decreases with age (Guzman & Kingston, 2012). In our study, age differences were not observed, which could be a result of sample size of some age groups, but also a result of not taking into account the training experience as a control variable. But, also, these results could indicate additional validity of the questionnaire — i.e., that it can be applied to young athletes who play team sports of different ages, those who are in the period of early adolescence, middle adolescence, and entering late adolescence.

Regarding gender differences, following some previous research (Chin et al., 2012; Miller, 2000; Recours et al., 2004), we found that girls achieved lower scores on extrinsic motivation compared to boys, while no differences were recorded for other types of motivation. The obtained differences indicate the different socialization of boys and girls within sports: that boys are more oriented towards an ego-oriented approach to sports compared to girls, who are more oriented towards teamwork, building a healthy body, and not so much towards winning. The highlighted results are additionally supported by research that has shown that girls are more task-oriented than ego-oriented (Chin et al., 2012) and that they generally assess the motivational climate in the team as focused on learning and not on achievement (Vazou et al., 2006).

This study was one of the first in Serbia to address the motivation for sports in youth athletes who train and are on a developmental sports path in organized sports. As the results suggested, young athletes engaged in collective sports achieve higher scores on more autonomous types of motivation defined by SDT, such as intrinsic motivation, integrated motivation, and identified motivation. In line with SDT, athletes who have a developed identified motivation play sports to develop certain parts of the personality that they consider important. Combined with integrated motivation and intrinsic motivation, young Serbian athletes play sports because doing so reflects their essential lives and personal values, and because they want to develop new skills, become more competent, and enjoy the process. These results posit a healthy foundation for the further development of young athletes towards professionalism.

The results have practical application for psychologists in sports in the form of identifying the motivational profile of young athletes who are engaged in team sports in order to prevent the development of amotivation and to recognize low-autonomous forms of motivation in order to prevent dropping out from sports or burnout syndrome. In addition, the adaptation of the motivation assessment questionnaire for the Serbian sample of young athletes can be useful in working with the athletes themselves in order to better understand the internal factors that affect participation in sports and sport achievement; and in working with coaches as a guideline on how to change their approach with athletes who are at risk to develop amotivation.

In addition to the scientific contribution in the form of expanding the empirical results of sports psychology in Serbia, the results invite researchers in Serbia to further examine and test the translated questionnaire in order to create a more precise instrument for assessing motivation for participation in youth sports.

## Conclusion

The Sport Motivation Scale II - SMS-2 is one of the most widely used questionnaires of motivation towards sport participation, which is based on the theory of self-determination. As such, it found its role in researching the numerous processes in sport.

Until now, there has been no research that specifically dealt with the construct validity of this questionnaire on young athletes between the ages of 12 and 16 who are engaged in organized indoor collective (team) sports. In the sample of young Serbian athletes, it was shown that the SMS-2 has somewhat satisfactory psychometric characteristics, and six factors or types of motivation defined by the authors in the original questionnaire can be distinguished. The translation of the questionnaire from English to Serbian proved to be valid with respect to gender, age, and type of sport. It is also essential to highlight that the present study was conducted on a sample of young athletes who are engaged in organized team sports, not recreational sports or school sports.

Future research should explore the psychometric characteristics of the employed questionnaire in a sample of Serbian athletes competing in individual sports and take into account the effect of years of training and competition on motivation to play sports. To gain a better insight into age trends of motivation for sports, longitudinal studies should be conducted. Better understanding of age trends in motivation is an important topic, keeping in mind that young athletes aged 13 to 16 are at risk of dropping out of sports. Continued research of motivation is needed to develop interventions and preventive activities so that young athletes remain in sports and achieve their sporting potential.

## Limitations

The potential limitations of this research are the absence of athletes who compete in individual sports as well as the conditions in which athletes completed the questionnaire. More precisely, some athletes filled out the questionnaire right after practice or just before practice, and some had to do it in the dressing room. These conditions could have affected some of the responses, considering that the athletes were very limited in time. Also, it should be noted that the convergent and discriminant validity of the adaptation has not been calculated, as well as test-retest reliability. Years participating in official competitions were not controlled in the study.
